# Structural and Biochemical Insights into the Peptidoglycan Hydrolase Domain of FlgJ from *Salmonella typhimurium*

**DOI:** 10.1371/journal.pone.0149204

**Published:** 2016-02-12

**Authors:** Patryk Zaloba, Ben A. Bailey-Elkin, Miriam Derksen, Brian L. Mark

**Affiliations:** Department of Microbiology, University of Manitoba, Winnipeg, Canada; Russian Academy of Sciences, Institute for Biological Instrumentation, RUSSIAN FEDERATION

## Abstract

FlgJ is a glycoside hydrolase (GH) enzyme belonging to the Carbohydrate Active enZyme (CAZy) family GH73. It facilitates passage of the bacterial flagellum through the peptidoglycan (PG) layer by cleaving the β-1,4 glycosidic bond between *N*-acetylglucosamine and *N*-acetylmuramic acid sugars that comprise the glycan strands of PG. Here we describe the crystal structure of the GH domain of FlgJ from bacterial pathogen *Salmonella typhimurium* (*St*FlgJ). Interestingly, the active site of *St*FlgJ was blocked by the C-terminal α-helix of a neighbouring symmetry mate and a β-hairpin containing the putative catalytic glutamic acid residue Glu223 was poorly resolved and could not be completely modeled into the electron density, suggesting it is flexible. Previous reports have shown that the GH73 enzyme Auto from *Listeria monocytogenes* is inhibited by an N-terminal α-helix that may occlude the active site in similar fashion. To investigate if the C-terminus of *St*FlgJ inhibits GH activity, the glycolytic activity of *St*FlgJ was assessed with and without the C-terminal α-helix. The GH activity of *St*FlgJ was unaffected by the presence or absence of the α-helix, suggesting it is not involved in regulating activity. Removal of the C-terminal α-helix did, however, allow a crystal structure of the domain to be obtained where the flexible β-hairpin containing residue Glu223 was entirely resolved. The β-hairpin was positioned such that the active site groove was fully solvent-exposed, placing Glu223 nearly 21.6 Å away from the putative general acid/base residue Glu184, which is too far apart for these two residues to coordinate glycosidic bond hydrolysis. The mobile nature of the *St*FlgJ β-hairpin is consistent with structural studies of related GH73 enzymes, suggesting that a dynamic active site may be common to many GH73 enzymes, in which the active site opens to capture substrate and then closes to correctly orient active site residues for catalysis.

## Introduction

Glycoside hydrolase (GH) enzymes have been classified into families based on primary amino acid sequence (http://www.cazy.org/) [1]. Members of GH family 73 (GH73) are β-N-acetylglucosaminidases that cleave the β-1,4 glycosidic linkage between the N-acetylglucosamine (GlcNAc) and N-acetylmuramic acid (MurNAc) residues of bacterial cell wall peptidoglycan (PG) [2–6]. Bacterial growth, division, flagella formation, sporulation, protein secretion and other important processes depend on the controlled remodelling of PG [7,8] which is carried out, in part, by GH73 β-N-acetylglucosaminidases [8–12]. In some instances, GH73 enzymes are associated with bacterial pathogenicity, for example, LytB being critical for pneumococcal nasal colonization and cell division [13], Auto for pathogenic listeriosis caused by *Listeria monocytogenes* [10], and FlgJ for biogenesis and function of the flagellum, which is a major virulence factor of motile pathogenic bacteria [14].

Most GH enzymes use two enzymatic carboxyl groups in a catalytic reaction that proceeds through one of two mechanisms. The first mechanism involves two-steps where, initially, one carboxyl group acts as a general acid to protonate the glycosidic oxygen while the other acts as a nucleophile that attacks the anomeric carbon of the glycosidic bond. This combined action cleaves the glycosidic bond and results in the formation of a glycosyl-enzyme intermediate. Subsequently, the carboxyl group that initially protonated the glycosidic oxygen acts as a general base to activate a water that attacks the anomeric center of the intermediate and yields a product with retained anomeric stereochemistry [15,16]. In the second, single-step mechanism, one carboxyl group acts as a general acid to protonate the glycosidic oxygen while the other acts as a general base to activate an intervening water molecule that attacks the anomeric carbon of the glycosidic bond, resulting in bond cleavage and a product with inverted anomeric stereochemistry [15,16]. Some GH enzymes use a third mechanism, that employ a two-step configuration-retaining mechanism that exploits the presence of a neighbouring 2-acetamido group on the substrate, as first predicted by Koshland [17]. In this case, the 2-acetamido group acts as a nucleophile in place of an enzyme carboxyl group; this process is known as substrate-assisted catalysis (SAC; for a comprehensive review of glycosidase mechanisms see [18]).

Previous studies have identified an invariant glutamate in GH73 enzymes that likely acts as the general acid catalyst protonating the glycosidic bond in the above mechanisms [2,4,5,19–22]. However, identification of a second conserved active site carboxylate remains elusive and it appears some GH73 enzymes may use substrate-assisted catalysis as opposed to the classical mechanisms that require two enzymatic carboxyl groups [5,19,20]. Additionally, it is unclear whether GH73 enzymes with two catalytic carboxylate-carrying residues proceed through a configuration retaining or inverting mechanism. Characterized examples of GH73 enzymes with two identified carboxylate residues that are currently predicted to use an inverting mechanism include FlgJ, Auto and TM06330 [2,4,5,21], while those presumably lacking a second catalytic carboxylate residue, and predicted to use a SAC mechanism, include the autolysins Atl_WM_, AcmA, and LytB [5,19,20].

To explore the structural basis of FlgJ activity, we determined the crystal structure of the FlgJ GH domain from *S*. *typhimurium* (*St*FlgJ) to 1.8 Å resolution. Interestingly, the active site of the domain was occupied by a C-terminal α-helix of a neighbouring symmetry mate. Previous reports have described similarly occluded active sites in the crystal structure of the GH73 enzyme Auto, a virulence factor of *L*. *monocytogenes*, as well as in the crystal structure of the *Sphingomonas sp*. FlgJ enzyme (*Sp*FlgJ) [2,23]. The active sites of Auto and *Sp*FlgJ were occupied *in trans* by an N-terminal α-helix and C-terminal strand, respectively, from neighbouring monomers within the crystal lattice. Furthermore, the N-terminal α-helix of Auto was previously found to inhibit glycolytic activity, and necessitated proteolytic removal in order to activate the domain [2]. The discovery of three GH73 enzymes (Auto, *Sp*FlgJ and *St*FlgJ) with occluded active sites led us to hypothesize that autoinhibition by N- or C-terminal motifs may control the activity of many GH73 enzymes.

To investigate whether the C-terminus of *St*FlgJ regulates activity in the same way as the N-terminus of Auto, a related GH73 enzyme, the glycolytic activity of *St*FlgJ was measured in the presence and absence of the C-terminal α-helix using zymogram assays. Contrary to our hypothesis, we found the catalytic activity of *St*FlgJ to be unaffected by presence or absence of its C-terminal α-helix, suggesting the helix does not regulate function. Removing the C-terminal α-helix, however, allowed us to determine a 2.1 Å crystal structure of the domain in which the extended β-hairpin, known to accommodate a second putative catalytic carboxyl group, was well ordered and revealed that the active site of *St*FlgJ seems capable of opening widely, possibly aiding in substrate capture and turnover.

## Materials and Methods

### Expression, purification and crystallization of GH73 enzyme *St*FlgJ

The open reading frame for the C-terminal glycoside hydrolase domain of *St*FlgJ (residues 151–316; NCBI Gene ID: 1252700) was amplified by PCR from genomic DNA isolated from *Salmonella enterica subsp*. *enterica serovar Typhimurium str*. *LT2* (B) using primers 5’-GATATACATATGGACAGTAAAGACTTTCTGGCC-3’ and 5’-GATATAGGATCCAAAGAGATTGTCGAGATTCGC-3’. The primers introduced NdeI and BamHI restriction sites at the 5’ and 3’ ends of the PCR amplicon, respectively, to facilitate ligation of the DNA into a modified pET28b(+) vector that fuses a His_6_ purification tag in-frame to the 3’-end of the GH domain open reading frame. The resulting expression vector, pET28-*St*FlgJ_GH(151–316)_, was used to transform *Escherichia coli* BL21 Gold (DE3) cells. The cells were grown at 37°C to an optical density (OD_600nm_) of 0.4–0.6 in 500 mL LB medium supplemented with 35 μg/mL kanamycin (Fisher). The culture temperature was subsequently lowered to 28°C, and recombinant protein expression induced with the addition of 1 mM isopropyl-D-thiogalactoside (IPTG) (Invitrogen) for 4 hours with shaking. Cells were pelleted by centrifugation (3450 x g, 20 min at 4°C) and either stored at -80°C or immediately re-suspended in 20 mL ice cold lysis buffer (1M NaCl, 50 mM Tris-HCl pH 8.0, 0.1 mM phenylmethylsulfonyl fluoride) for cell disruption. Cells were lysed by French press (Aminco) and the soluble fraction recovered by centrifugation (17200 x g, 30 min at 4°C). *St*FlgJ_GH(151–316)_ was isolated by affinity chromatography using Ni-NTA resin (Qiagen) pre-equilibrated with the lysis buffer. Recombinant protein was bound to the resin over a 20 min incubation period with agitation on ice and transferred to a column for thorough washing with lysis buffer (1M NaCl, 50 mM Tris-HCl pH 8.0), which was also used as a binding buffer. *St*FlgJ_GH(151–316)_ was eluted using 750 mM NaCl, 37.5 mM Tris-HCl pH 8.0, 250 mM imidazole. The eluted protein was dialyzed overnight against 800 mM NaCl, 10 mM Tris-HCl pH 8.0; and once more the following day against 500 mM NaCl, 10 mM Tris-HCl pH 8.0. The dialyzed protein was concentrated and purified using an FPLC (ÄKTA) and HiLoad 16/60 Superdex 75 size exclusion column (GE Healthcare) equilibrated with 500 mM NaCl 10 mM Tris-HCl pH 8.0. Fractions containing *St*FlgJ_GH(151–316)_ were pooled, concentrated and subjected to crystallization trials.

To remove the 15 amino acids from the C-terminus in the *St*FlgJ_GH(151–316)_ GH domain found to be blocking the domain active site *in trans*, the plasmid pET28-*St*FlgJ_GH(151–316)_ was redesigned using a new primer set (5’GATATACATATGGACAGTAAAGACTTTCTGGCC-3’ and 5’-GATATAGGATCCACTCATCGCTTTCAACTGCTG-3’, sense and antisense, respectively) which amplified a sequence that excluded the last 15 codons of the full-length domain and placed a BamHI site at the 3’end. The modified *St*FlgJ amplicon, encoding residues 151–301 of FlgJ, was ligated into the pET28b(+) vector described above to generate pET28-*St*FlgJ_GH(151–301)_, from which protein was expressed and purified as describe above for *St*FlgJ_GH(151–316)_.

Crystallization of *St*FlgJ_GH(151–316)_ catalytic domain was performed at 20°C using the hanging drop vapor diffusion method. Crystal were grown within 72 hours using 2 μL of protein solution at 35 mg/mL in 500 mM NaCl 10 mM Tris-HCl pH 8.0 mixed with an equal volume of mother liquor consisting of 18–22% (w/v) polyethylene glycol 3350 and 250–350 mM NaI. The drops were equilibrated by vapour diffusion against 150 μL of mother liquor. Crystals grew to maximum dimensions of ~0.1 x 0.1 x 1 mm. *St*FlgJ_GH(151–301)_ and *St*FlgJ_GH(151–316)_ crystalized under identical conditions, albeit in different space groups.

### X-ray data collection, processing and structure determination

Crystals were harvested by sweeping through a solution containing 22% (w/v) PEG 3350, 350 mM NaI, 15% (v/v) glycerol, before being flash-cooled in liquid nitrogen. X-ray diffraction data were collected in-house at 93K from individual crystals using a MicroMax-007 HF X-ray source and R-axis 4++ detector (Rigaku). X-ray data were integrated using iMosflm and scaled and merged using Aimless within the CCP4 software suit [24]. Both *St*FlgJ_GH(151–316)_ and *St*FlgJ_GH(151–301)_ crystals were grown in the presence of iodide and initial phase estimates could be determined using a single wavelength anomalous dispersion (SAD) phasing experiment, using an in-house Cu K-α X-ray source. Iodide substructures used for phasing diffraction data were identified using phenix.autosol [25]. All models were built using Coot [26], and refinement was carried out using phenix.refine [25].

### FlgJ Activity Assay

To determine whether the C-terminus of the *St*FlgJ GH domain auto-inhibited catalytic activity in solution, a zymogram activity assay based on a previously described approach [2] was carried out on *St*FlgJ_GH(151–316)_, *St*FlgJ_GH(151–301)_, as well as a catalytic mutant of *St*FlgJ_GH(151–316)_ (E223Q) [4]. The E223Q mutation was introduced into *St*FlgJ_GH(151–316)_ according to a modified version of [27]. Briefly, the *St*FlgJ_GH(151–316)_ open reading frame was amplified from pET28-*St*FlgJ_GH(151–316)_ using Phusion HF (NEB) and primers 5’ P-CAATACGAAAATGGCGAAGCG 3’ and 5’ P- AGTGGTGGTGATCTCCGTCACC -3’ (IDT). The PCR amplicon was digested with Dpn1 to remove methylated template plasmid, gel-extracted (Qiagen), re-circularized (NEB Quick Ligase) and transformed into *E*. *coli* DH5α. Plasmid was isolated and presence of the mutation was verified by DNA sequencing (The Centre for Applied Genomics, The Hospital for Sick Children, Toronto).

*E*. *coli* BL21-(DE3) Gold transformed with plasmid expressing either S*t*FlgJ_GH(151–316)_, *St*FlgJ_GH(151–301)_ or *St*FlgJ_GH(151–316)_(E223Q) were grown overnight in 3 mL LB containing 35 μg/ml kanamycin, subcultured into 65 ml of LB/kanamycin and grown at 37°C to OD_600_ = 0.50, whereupon protein expression was induced with 1mM IPTG for 3.5 hours at 28°C. Cultures were pelleted and stored at -80°C before lysis by sonication (Brandson) in 10 mL 1M NaCl, 50mM Tris pH8, 1 mM PMSF. The expressed FlgJ hydrolase domains were then affinity purified from clarified lysates as described above.

The zymogram gel was prepared as previously described (15% acrylamide gel containing 0.2% *M*. *lysodeikticus* lyophilized cells (Sigma), but no sodium dodecyl sulfate (SDS) [2]. Protein samples were mixed with sample loading buffer lacking SDS and β-mercaptoethanol and not boiled prior to gel loading to prevent denaturation. Following electrophoresis, the zymogram gel was rinsed with Milli-Q H_2_O, prestained with 100 mM NaAcetate, pH 4.8 and stained with 0.1%Methylene Blue, 0.01% KOH as previously described and destained in Milli-Q H_2_O overnight [2]. Standard SDS-PAGE analysis on a 12% acrylamide gel was carried out to assess protein purity.

## Results and Discussion

**Fig 1:**
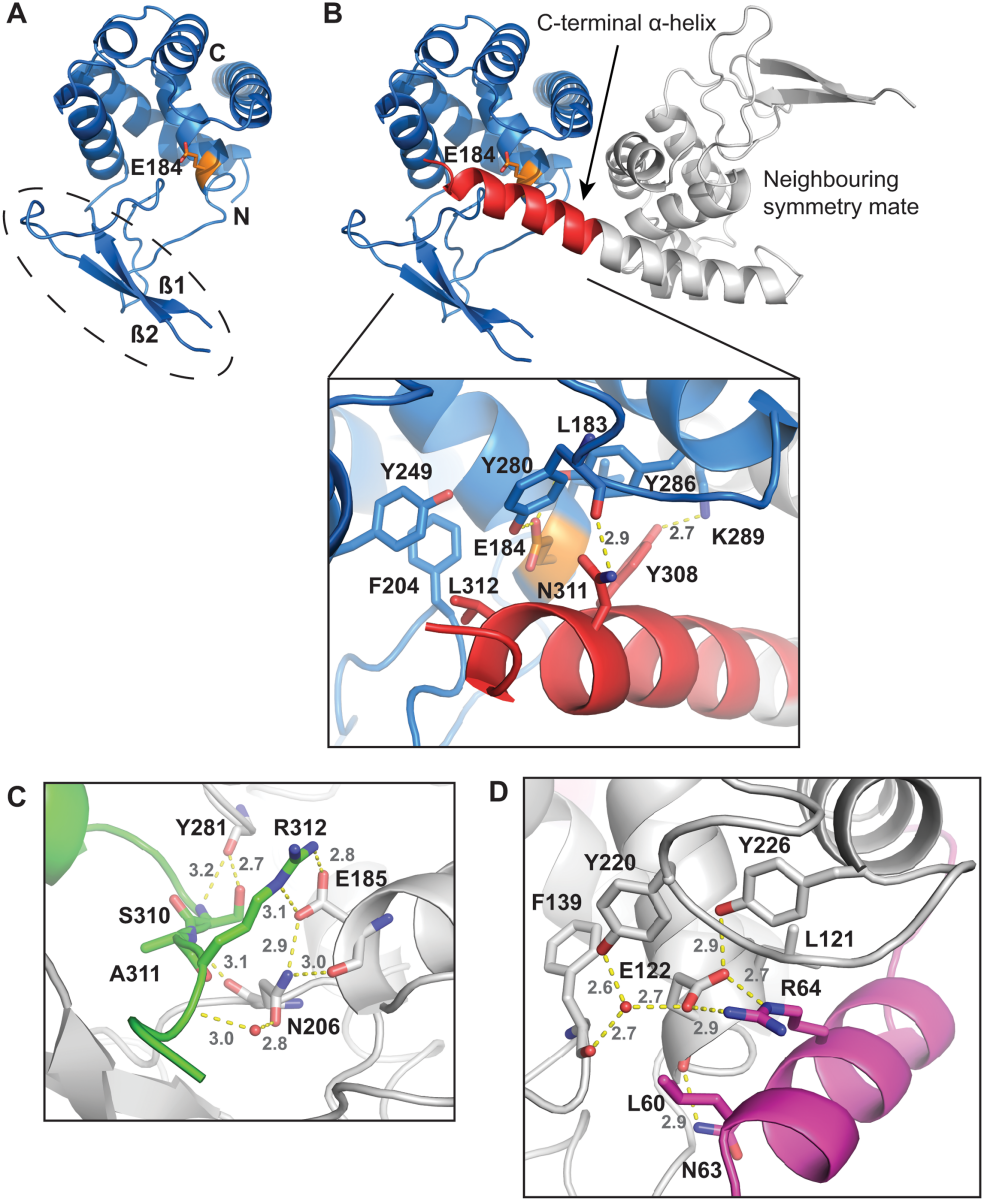
Structural description of *St*FlgJ_GH(151–316)_. A) Cartoon diagram of the FlgJ GH domain from *Salmonella typhimurium* (*St*FlgJ_GH(151–316)_) (blue) is shown with the catalytic glutamate (E184; orange), within the active site groove. Circled is the incomplete β-hairpin, with missing residues 223–229, that forms half of the active site groove. B) All molecules within the crystal structure were found to have their active sites blocked *in trans* by the C-terminal α-helix (red) of a neighbouring symmetry mate (grey) (conserved catalytic residue E184 is orange). C) *In trans* blockage of the GH domain active site of *Sp*FlgJ (grey carbons) by the C-terminus of a symmetry mate (green carbons) [21,23]. D) *In trans* blockage of the GH domain active site of Auto (grey carbons) by the N-terminal inhibitory helix of a symmetry mate (magenta carbons) [2]. Structural models were generated using PyMol [28].

The crystal structure *St*FlgJ_GH(151–316_ had a single molecule comprising the asymmetric unit and was refined to 1.8 Å resolution (Table 1). The domain was found to adopt a lysozyme-like fold common to other GH73 enzymes, which consists of an α-helical subdomain packed against a β-lobe that forms an extended β-hairpin motif [2,5,23] (Fig 1A). The α-helical domain and β-hairpin of *St*FlgJ_GH(151–316)_ together form a groove-shaped active site, with the α-helical subdomain containing an α-central helix that houses a conserved glutamate general acid/base (E184), and the β-hairpin, in turn, containing a second glutamate residue (E223) previously determined to be required for GH activity [4]. Residues 223–229—which form a loop connecting the two strands of the β-hairpin motif—were disordered and could not be modeled. Similar results have been reported for the crystal structures of other GH73 enzymes, indicating that the β-hairpin is likely mobile in this family of enzymes [5,21–23].

**Table 1:** Crystallographic data and model refinement statistics.

Crystal	*St*FlgJ_GH(151–316)_	*St*FlgJ_GH(151–301)_
**Crystal geometry**		
Space group	P 2_1_ 2_1_ 2_1_	C 2
Unit-cell dimensions (Å, °)	a = 38.8, b = 43.6, c = 107.9, α = β = γ = 90	a = 105.7, b = 61.1, c = 65.2, α = 90, β = 106.7, γ = 90
**Crystallographic data**		
Wavelength (Å)	1.54187	1.54187
Resolution Range (Å)^a^	31.50–1.80 (1.86–1.80)	37.47–2.15 (2.22–2.15)
Total observations^a^	235307 (12749)	159127 (13542)
Unique reflections^a^	17650 (997)	21763 (1867)
Multiplicity^a^	13.3 (12.8)	7.3 (7.3)
Completeness (%)^a^	99.8 (98.4)	99.7 (99.0)
Anomalous completeness (%)^a^	99.8 (98.2)	99.8 (99.1)
Mean I/σ(I)^a^	12.9(3.0)	10.3 (2.5)
*R*_merge_ (%)^a^	11.4 (68.9)	15.1 (82.7)
CC1/2^a^	0.996 (0.959)	0.994 (0.724)
Wilson B-factor (Å^2^)	18.6	15.9
**Phasing statistics**		
Heavy atom sites (Iodide)	11	10
Figure of merit	0.55	0.44
Figure of merit after RESOLVE	0.75	0.77
**Refinement statistics**		
*R*_work_/*R*_free_ (%)	18.2/21.6	17.5/25.2
Reflections in test set	900	1096
Protein atoms	1174	3250
Ligands	12	13
Water	150	177
**Average *B*-factor (Å**^**2**^**)**		
Protein	29.8	33.8
Ligands	32.3	32.0
Solvent	38.2	33.2
**Root mean square deviations**		
Bond lengths/angles (Å/°)	0.011/1.27	0.014/1.41
**Ramachandran plot**		
Favoured/allowed (%)	99.40/0.60	94.30/5.52
**PDB entry**	5DN4	5DN5

^a^ Values in parentheses are for the highest resolution shell.

**Fig 2:**
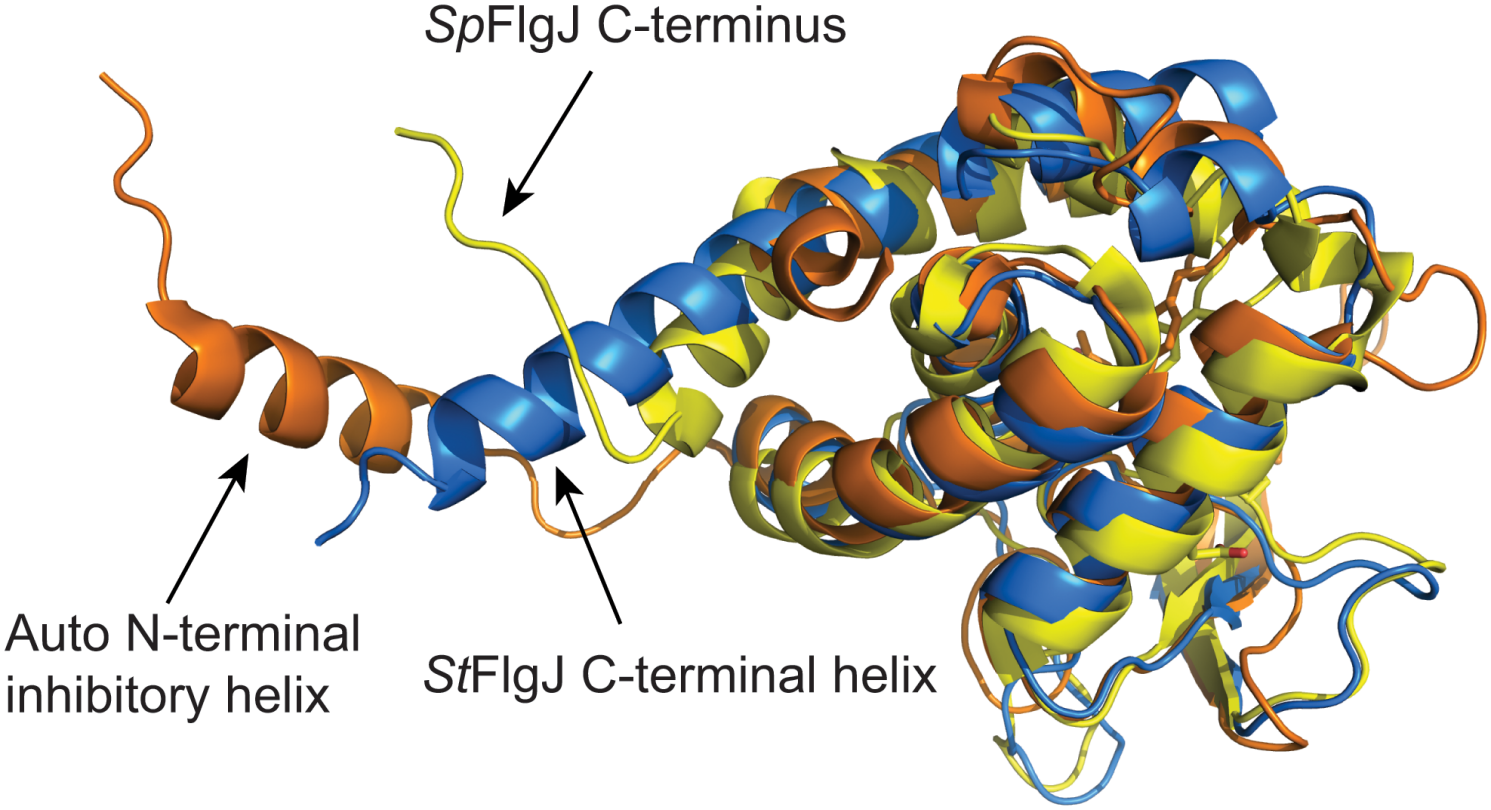
Spatial conservation of the N- and C-termini of GH73 enzymes. A superposition of *St*FlgJ_GH(151–316)_ (PDB: 5DN4; blue) with Auto (PDB: 3FI7; orange) and FlgJ from *Sphingomonas* sp., *Sp*FlgJ (PDB: 2ZYC; yellow), reveal that the N- and C-terminal regions of the enzymes are spatially conserved. Auto and *Sp*FlgJ share high structural similarity to *St*FlgJ_GH(151–316)_ with RMSD values of 2.2 Å and 3.6 Å, respectively. For Auto and *Sp*FlgJ, 133 and 154 C_α_ positions were aligned to *St*FlgJ_GH(151–316)_, respectively. All superpositions were generated using the DaliLight server [29].

Interestingly, the active site of *St*FlgJ_GH(151–316)_ was found to be occupied by the C-terminal α-helix of a neighbouring symmetry mate (Fig 1B). The C-terminal α-helix *St*FlgJ_GH(151–316)_ packs into the active site of the symmetry mate via hydrophobic and hydrogen-bonding interactions. Hydrophobic interactions were found to occur between L312 of the helix and F204 and Y249 of the active site of the symmetry mate, as well as between Y308 of the helix and L183 and Y286 of the symmetry mate active site. Hydrogen bonds occur between Y308 and N311 of the helix and K289 and backbone amide of Y280 of the symmetry mate active site. A similar in *trans* blockage of the GH domain active site of *Sp*FlgJ has also been observed, where the C-terminus extends into the active site of its neighbour [21,23] (Fig 1C). Unlike *St*FlgJ, the C-terminal extension of *Sp*FlgJ forms extensive hydrogen-bonding interactions with the active site of the symmetry mate that includes the putative general acid/base residue E185, which forms a salt bridge and hydrogen bond with R312 of the C-terminal extension. The observed active site occlusion in these FlgJ proteins is reminiscent of the N-terminal α-helix of the *L*. *monocytogenes* GH73 enzyme Auto, which also blocks the active site *in trans* in the crystalline state [2]. As for *Sp*FlgJ (Fig 1C), an arginine (R64) on the N-terminal α-helix of Auto forms a salt bridge and hydrogen bond with the active site general acid base E122 of the enzyme (Fig 1D). It should be noted that Bublitz *et al*. found it necessary to truncate the N-terminus of Auto to achieve crystallization of the protein, and the *in trans* obstruction of the Auto active site in the crystal structure was suggested to be non-physiological, since the protein is monomeric in solution [2]. However, they also proposed that in solution, the natural, full-length N-terminus blocks the active site of Auto *in cis* and they demonstrated that it must be removed proteolytically for the enzyme to become activated [2]. A superposition of the *St*FlgJ and *Sp*FlgJ GH domain structures with Auto reveals that the C- and N-termini of these enzymes are spatially conserved (Fig 2), which suggested that FlgJ might be auto-inhibited by the C-terminal α-helix, in similar fashion to the auto-inhibition of Auto by an N-terminal α-helix.

**Fig 3:**
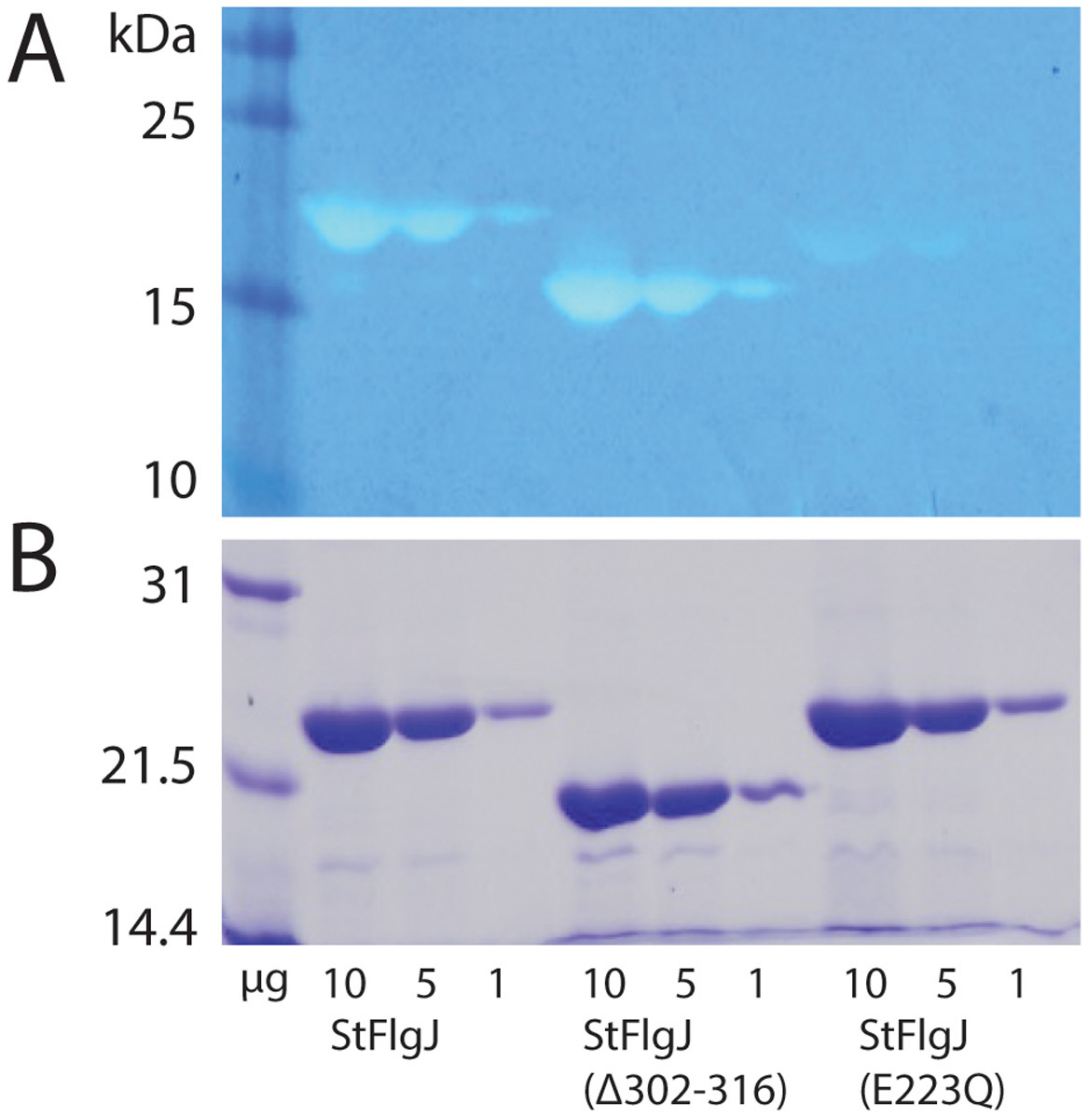
Activity assay. A) Zymogram activity assay of the full-length *St*FlgJ GH domain (*St*FlgJ (151–316)), the *St*FlgJ GH domain lacking the C-terminal α-helix (StFlgJ (Δ302–316)), a catalytic mutant of the full-length *St*FlgJ GH domain (StFlgJ (E223Q)). The proteins and the amount of each applied to the gel is shown in micrograms along the bottom of the figure. The presence or absence of the C-terminal helix did not affect *St*FlgJ GH domain activity; whereas, the previously described catalytic mutation E223Q [4] reduced activity as expected. B) SDS-PAGE analysis showing the purity and abundance of each protein sample used in the zymogram assay.

To determine whether the C-terminus of *St*FlgJ_GH(151–316)_ autoinhibits the enzyme in solution, the full-length *St*FlgJ GH domain, *St*FlgJ_GH(151–316)_ and a C-terminal truncated form of the domain (*St*FlgJ_GH(151–301)_) were tested for activity using a zymogram assay. Contrary to our hypothesis, the assays revealed that the *St*FlgJ GH domain is fully active both with and without the C-terminal α-helix, indicating that the helix does not auto-inhibit *St*FlgJ GH activity and is likely not functionally equivalent to the N-terminal auto-inhibitory helix of Auto (Fig 3). In contrast, mutating the putative catalytic residue E223 to glutamine in the beta-hairpin of *St*FlgJ_GH(151–316)_ reduced hydrolase activity to residual levels, which is consistent with previous work identifying E223 as a catalytic residue [4]. Given the C-terminus of *St*FlgJ did not block activity of the enzyme as we had anticipated, it may instead participate in coiled-coil interactions with other flagellar component proteins as previously proposed [30].

**Fig 4:**
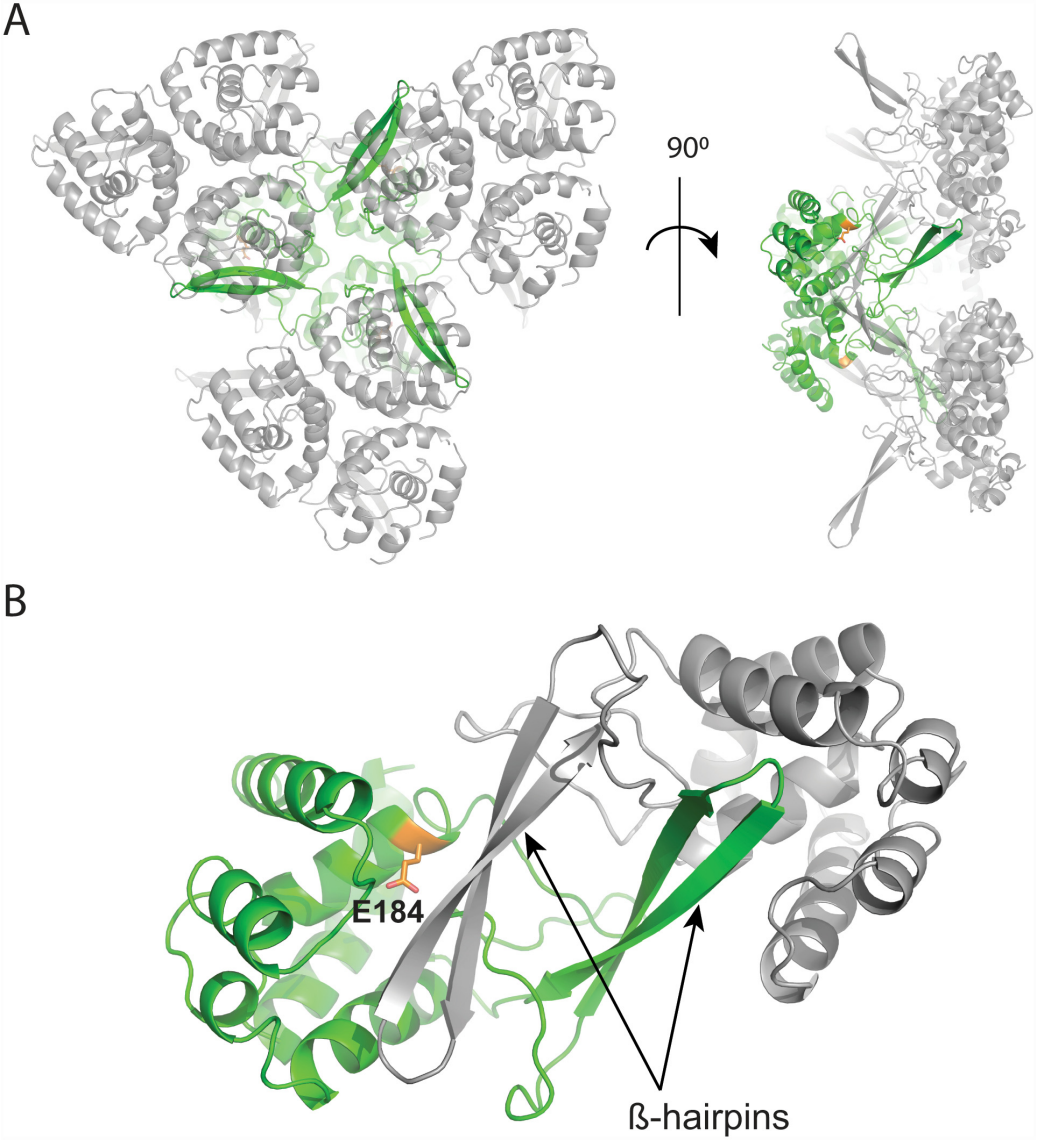
Crystal packing of *St*FlgJ_GH(151–301)_. A) Packing of asymmetric units (ASUs) within the crystal structure of the C-terminally truncated mutant, *St*FlgJ_GH(151–301)_. Each ASU is comprised of three copies of *St*FlgJ_GH(151–301)_. One ASU is coloured green with three neighbouring ASUs coloured grey for clarity. B) The β-hairpin of each monomer of each ASU packs identically against the active site of a monomer within a neighbouring symmetry related ASU (grey) within the crystal structure. This packing imparts order to β-hairpin of each monomer while it partly occluding the active site of each copy of the protein in the crystal (conserved active site residue E184 shown in orange).

**Fig 5:**
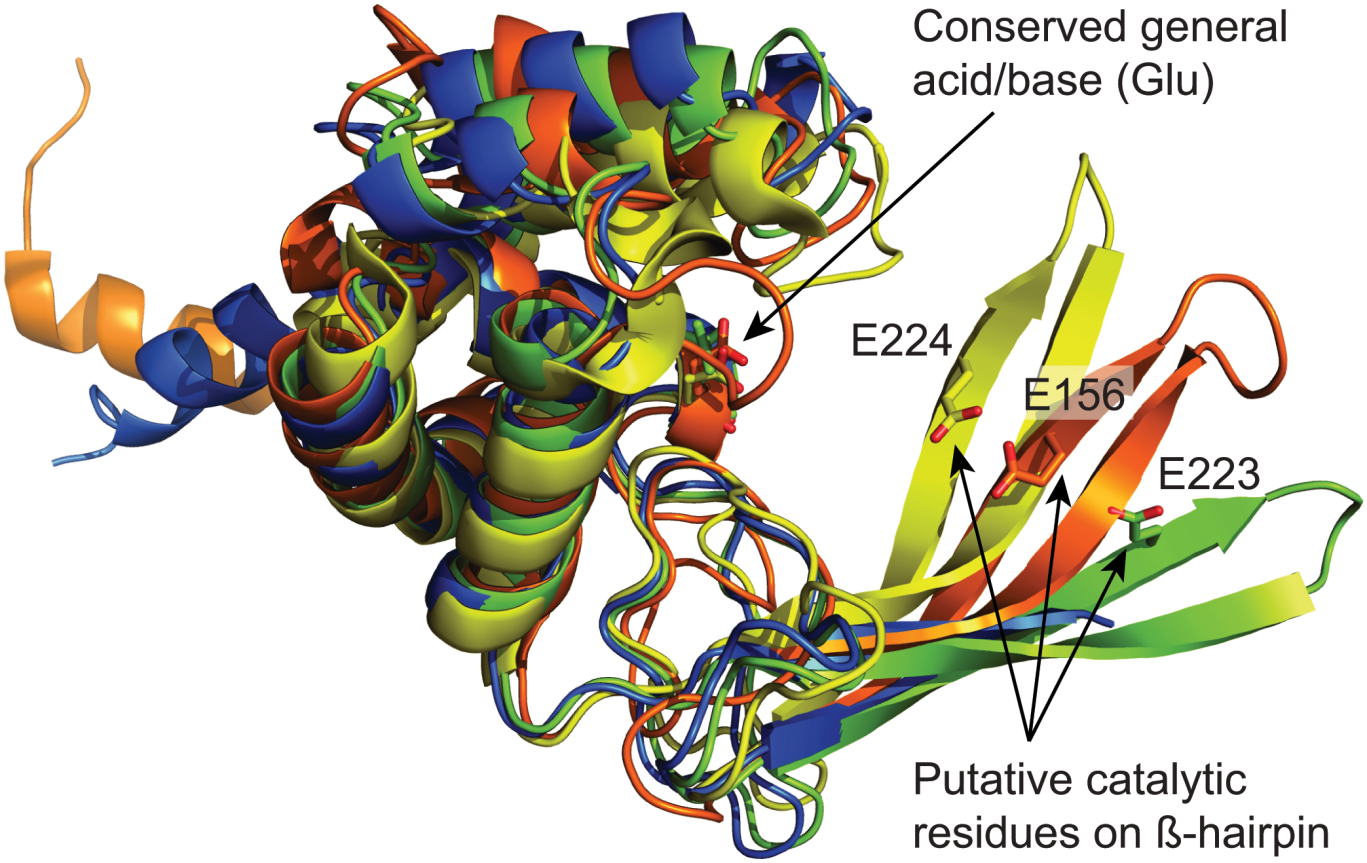
Structural comparison of GH73 enzymes. A superposition of *St*FlgJ_GH(151–301)_ (PDB: 5DN5; green), Auto (PDB: 3FI7; orange)[2] and *Sp*FlgJ (PDB: 3VWO; yellow). β-hairpin residues (E223 for *St*FlgJ_GH(151–301)_, E156 for Auto, and E224 for *Sp*FlgJ) are shown on each β-hairpin. Also superposed is the structure of *St*FlgJ_GH(151–316)_ (PDB: 5DN4; blue). Superposition using the DaliLight server [29] showed, for Auto: C_α_ rmsd of 3.3 Å over 140 equivalent positions to *St*FlgJ_GH(151–301)_; *Sp*FlgJ: C_α_ rmsd of 2.3 Å over 140 equivalent positions to *St*FlgJ_GH(151–301)_.

Next, we determined the crystal structure of the truncated GH domain (*St*FlgJ_GH(151–301)_) to 2.2 Å resolution (Table 1) to study the active site of *St*FlgJ in the absence of the C-terminal α-helix. The asymmetric unit contained three copies of the truncated domain (Fig 4A). Though the C-terminal α-helix no longer occupied the active site, the catalytic β-hairpin of each domain was found to partly occlude the active site of neighbouring domains in the crystal structure (Fig 4B). While this crystal packing did not provide an unobstructed active site, it did impart significant structural order to the β-hairpin. The β-hairpin was found to be located further from the helical domain of the enzyme than any previously reported structure of a GH73 enzyme, including Auto and *Sp*FlgJ (Fig 5). A superposition of *St*FlgJ with the structures of other GH73 enzymes reveals substantial differences in the position of the β-hairpin harboring the putative catalytic E223 residue (Fig 5).

FlgJ [4,9,21], Auto [2], and the recently characterized GH73 enzyme from *Thermotoga maritima* TM0633 [5], all have a conserved glutamate on the β-hairpin that is required for catalysis, which suggests one of three possible catalytic mechanisms: 1) With substrate bound, the β-hairpin moves deep enough into the active site to allow the glutamate to act as a nucleophile, directly attacking the anomeric carbon of GlcNAc to promote glycosidic bond cleavage that proceeds via a glycosyl-enzyme intermediate where anomeric stereochemistry is retained; 2) The hairpin does not move deep enough into the active site to allow the glutamate to directly attack GlcNAc but instead positions the glutamate to activate an intervening water molecule that, in turn, attacks the anomeric carbon of GlcNAc to promote glycoside bond cleavage with inversion of anomeric configuration; or finally 3) The β-hairpin moves to position the glutamate so that it can help facilitate a substrate assisted catalytic mechanism whereby the 2-acetamido group of GlcNAc acts as the nucleophile to drive a reaction that proceeds via an oxazolinium ion intermediate and generates a product with retained stereochemistry. Current structural and functional studies point towards the inverting mechanism (#2 above) as the likely mechanism given the large distances (~10–20 Å) that have been observed between the glutamate general acid on the helical domain and putative general base on the β-hairpin of these enzymes. GH enzymes using an inverting mechanism typically have the general acid and general base ~10.5 Å apart, whereas, those using a retaining mechanism that proceeds through a glycosyl-enzyme intermediate place these residues ~5.5 Å apart [16]. Though a distance approaching ~5.5 Å has yet to be observed between the catalytic residues of a GH73 enzyme, given the apparent flexibility of the β-hairpin either mechanism remains possible and stereochemical outcome studies will be needed to rule out the latter.

Alternatively, GH18, GH20, GH56, and GH84 enzymes employ a second enzyme carboxyl group (independent of the general acid/base) that appears to activate the 2-acetamido group of the substrate in order to participate in a substrate-assisted reaction and help to stabilize the resulting oxazolinium ion intermediate (#3 above) [31–35]. The SAC mechanism of GH85 enzymes uses an asparagine in place of an enzyme carboxylate to facilitate 2-acetamido group participation [36]. While it is possible that the glutamate on the β-hairpin could serve as the second carboxyl group that engaging the 2-acetamido group in a SAC mechanism, enzymes that use a SAC mechanism typically have ridged active site architectures and a number of additional residues to help orient the carbonyl oxygen of the 2-acetamido within striking distance of the anomeric center of GlcNAc [37]. Evidence for these additional residues is lacking in *St*FlgJ, *Sp*FlgJ and Auto, which may make this mechanism less likely for these enzymes. Interestingly however, there exist additional groups of GH73 enzymes where the β-hairpin contains a carboxyl group that is not needed for catalysis, as in *Staphylococcus warneri* M Atl_WM_ [20]; in the GH73 enzyme AcmA from *Lactococcus lactis ssp*. *cremoris* MG1363, the β-hairpin contains a carboxyl group (Glu) that can be exchanged for an amide group (Gln) without complete loss of activity [19]. Additionally, some GH73 sequences appear to completely lack a suitable acidic residue within the β-hairpin [5]. These enzymes may indeed use a SAC mechanism that does not depend on the use of a second enzymic carboxyl group, but may instead use an alternative residue, such as an asparagine as found in GH85 enzymes. Further structural and functional studies, including the development of straightforward kinetic assays using substrate analogues, will be needed to resolve which catalytic mechanism(s) are employed by GH73 enzymes.
